# Supplementary material for: Towards hepatitis B elimination in Ghana: vaccination coverage and its predictors among informal sector workers in Kejetia, Kumasi, Ghana
Source: PLoS One. 2026 Apr 2;21(4):e0334205. doi: 10.1371/journal.pone.0334205 (PMC13046117; doi:10.1371/journal.pone.0334205)
Supplement: S1 File — (DOCX) [file pone.0334205.s001.docx]

KEJETIA VIRAL HEPATITIS

You have been invited in a research study that seeks to assess hepatitis B vaccination among sellers and buyers at Kejetia. Please note that by completing this questionnaire you are voluntarily agreeing to participate in this research study. You will remain anonymous and your data will be treated with great confidentiality at all times.

The researchers will be glad if you could complete the questionnaire in full. Please tick the appropriate box (es) and/or fill the blank spaces provided.

**1. Have you ever heard of hepatitis B infection?**

Yes

No

**2. If yes, what is your source of information on hepatitis B?**

Media

School

Church

Friends

Hospital

Other:

**3. Which group of people are encouraged to take the hepatitis B vaccine?**

Those who are infected with hepatitis B

Those who do not yet have the hepatitis B infection

Other:

**4. Have you taken Hepatitis B vaccine before?**

Yes

No

Other:

**5. If no, Reasons for not vaccinated**

Cost of the vaccination

Do not know where to go

Do not have any reason

Busy schedule

Not sick

Afraid of getting the infection from the vaccine

Fear of vaccine side effects

Do not think HBV vaccine is important

Do not know where to go to get HBV vaccine

I always forget to take it

Vaccine not available in my hospital

Needle phobia

Other:

**6. If no, (not vaccinated), will you take the hepatitis B vaccine if it were given free of charge?**

Yes

No

Other:

**7. If yes, how many doses of the hepatitis B vaccine have you taken?**

0

1

2

3

≥4

**8. What was your source of hepatitis B vaccination?**

Organised by Church

Organised by School

Organised by parents

By self initiative

Other:

**9. If vaccinated, when was the last time you took Hepatitis B vaccine?**

18. If vaccinated, do you have the document or yellow card showing proof of vaccination?

Yes

No

Other:

**10. Do you have a close person who has HBV infection?**

Yes

No

Other:

**11. Where you have stayed for greater part of your life, will you consider that place to be?**

Rural

Urban

Other:

**12. The hepatitis B testing and vaccination at the moment cost 60.00 for first shot and 30.00 for 2nd and third each; How expensive do you think is the testing and vaccination of Hepatitis B?**

Free

Reasonable

Somewhat expensive

Expensive

Don't know

Other:

**13. Have you received education or training on Hepatitis B before?**

Yes

No

Other:

**DEMOGRAPHICS**

**14. Sex**

Male

Female

**15. Age**

**………………..**

**16. Which region in Ghana do you come from? (your hometown)**

Upper West

Upper East

North East

Savannah

Northern

Oti

Bono East

Bono Ahafo

Ahafo

Western

Volta

Greater Accra

Central

Western North

Ashanti

Eastern

Other:

**17. Marital status**

Divorced

Single

Married

Widowed

Cohabiting

**18. Religion**

Islamic

Christian

Traditional

Other:

**19. Educational level**

None

Basic

Secondary

Certificate

Diploma

HND

1st Degree

Master's degree

PhD

**20. Participant ID**

………………………….

**21. Initials of investigator in the order of first, middle and surname (eg. MAO for Michael Agyemang Obeng)**

………………………………………………………..

**22. Do you know if there is vaccine available for hepatitis B?**

Yes

No

Other:

**23. How much do you know about hepatitis B infection?**

None

Very little

Some

Alot

**24. Which of the following are transmission routes of hepatitis?**

No idea

contaminated food and water

mother-to-child

shaking hands with infected persons

hugging

through sex

Other:

**25. Profession categorization for participants**

Tailors & seamstress

Butchers & Meat Sellers

Barbers and Hairdressers

Waste collectors and cleaners

Fish mongers

Market porters (Kayayei)

Drivers & Mates

Food Vendors

Mobile workers (Hawkers)

Buyers

Cloth and Textile Sellers

Other:
