# Supplementary material for: Towards hepatitis B elimination in Ghana: vaccination coverage and its predictors among informal sector workers in Kejetia, Kumasi, Ghana
Source: PLoS One. 2026 Apr 2;21(4):e0334205. doi: 10.1371/journal.pone.0334205 (PMC13046117; doi:10.1371/journal.pone.0334205)
Supplement: Attachment — Submitted filename: Response to Reviewers.docx [file pone.0334205.s004.docx]

**RESPONSE TO REVIEWERS**

Manuscript ID: **PONE-D-25-51851R1**

Title: **Towards hepatitis B elimination in Ghana: vaccination coverage and its predictors among informal sector workers in Kejetia, Kumasi, Ghana**

We sincerely thank the Academic Editor and reviewers for their constructive feedback, which has helped us improve the quality and clarity of the manuscript. We have carefully revised the manuscript and addressed all comments raised. All changes have been highlighted in the tracked version of the manuscript.

Below we provide point-by-point responses.

**Reviewer #1**

The authors have addressed most of the concerns raised satisfactorily however, these outstanding concerns need to be addressed.

**Comment 1:** In Lines 159 -164, though the authors attempted to describe how the sampling was done, it still lacks enough detail. For instance, how many occupational subgroups were sampled, what were the sizes of these subgroups, how many were sampled from each subgroup to make up the sample size of 809 stated?

**Response:** We thank the reviewer for this important observation. The sampling procedure section has now been expanded to provide clearer detail on the occupational subgroups included in the stratified sampling approach (See page 9, line 167-169, of the unmarked manuscript).

**Comment 2:** Discussion - lines 328- 336 - when the results on vaccination coverage were being compared to other population groups, it would be helpful if the authors could provide some possible reasons for the similarities/differences observed compared to the other studies especially where HCWs were concerned.

**Response:** We appreciate this suggestion. The discussion has been expanded to include possible explanations for the differences observed between informal sector workers and healthcare workers in terms of vaccination coverage.

We now explain that healthcare workers typically have greater occupational risk awareness, institutional vaccination policies, and easier access to vaccination programs, which likely contributes to their higher vaccination coverage compared with informal sector workers (See page 20, lines 342-349 of the unmarked manuscript).

**Comment 3:** Figure labels - The axes of the figures need to be labelled well. For instance, the x-axis of Figure 1A could be labelled as Hepatitis B vaccination status and 1B could be Number of doses of Hepatitis B vaccine received

**Response:** We thank the reviewer for this observation. The figure labels have been revised to improve clarity.

**Reviewer #2**

The manuscript has been revised and could be published. However, the referencing seems problematic in some parts of the manuscript. I think that is a decision of the journal.

**Response:** We thank the reviewer for this observation. The reference list has been carefully reviewed and corrected where necessary.

We are grateful to the Academic Editor and the Reviewers for the constructive guidance, which has improved the clarity, rigor, and transparency of our manuscript. We believe that the revised version adequately addresses all concerns raised and now meets PLOS ONE’s publication criteria. We respectfully submit the revised manuscript for further consideration. Thank you.
